# Seasonal Differences in Light Exposure and the Associations With Health and Well-Being in Older Adults: An Exploratory Study

**DOI:** 10.1177/1937586717697650

**Published:** 2017-03-15

**Authors:** Amanda Nioi, Jenny Roe, Alan Gow, David McNair, Peter Aspinall

**Affiliations:** 1 Department of Human Health, School of Engineering and Physical Science, Heriot-Watt University, Edinburgh, UK; 2 School of Energy, Geoscience, Infrastructure and Society, Heriot-Watt University, Edinburgh, UK; 3 Center for Design and Health, School of Architecture, University of Virginia, Charlottesville, VA, USA; 4 Stockholm Environment Institute, University of York, York, UK; 5 School of Management and Languages, Heriot-Watt University, Edinburgh, UK; 6 Centre for Cognitive Epidemiology, University of Edinburgh, Edinburgh, UK; 7 Dementia Centre, Hammond Care, Sydney, Australia; 8 School of Energy, Geoscience, Infrastructure and Society, Heriot-Watt University, Edinburgh, UK

**Keywords:** daylight, sleep/wake, circadian rhythms, physical activity, well-being, older adults, care home, visual function

## Abstract

**Objective:**

This article reports summer verses winter seasonal variations across a suite of blue light, illuminance levels and health and well-being indicators.

**Background:**

The quality of lighting in care homes has been assessed previously, yet seasonal comparisons and the associations with sleep quality are limited. This exploratory study investigates light exposure in two seasons to determine the changes over time and the associations with health and well-being.

**Methods:**

In a repeated measures design, 16 older people (aged 72–99 years) living in a care home had their personal light exposure and sleep/wake patterns monitored for 4 days. Cognitive ability, mental well-being, daytime physical activity, and visual function were assessed. Mean light levels at preset times across the day, duration in light exposure over 1,000 lux, and sleep parameters were computed. Statistical investigations included correlations exploring associations and paired means tests to detect the changes between seasons.

**Results:**

The mean morning illuminance level in summer was 466 lux and 65 lux in winter. Duration in bright light over 1,000 lux was 46 min in summer and 3 min in winter. Light measures were significantly higher in summer. There was no statistical difference in sleep quality parameters between seasons, but there were significant difference in daytime physical activity level (i.e., this was higher in summer).

**Conclusion:**

The findings indicate low level of light exposures experienced in both seasons, with exposure levels being particularly low in winter. This provides new insights into the limited amount of light older people receive independent of season and the possible impacts on sleep and daytime physical activity level.

## Background

Humans have evolved with exposure to a diurnal cycle of bright light from daylight to darkness, keeping circadian rhythms in synchronicity. Exposure to sufficient light has been reported as the strongest cue in regulating and synchronizing circadian rhythmicity (Foster, 2010). Light exposure for circadian synchronization is likely to differ with age, Turner and Mainster (2008) suggested that a person at age 75 requires 3 times as much light as that of a 45-year-old to elicit the same circadian response. It is also known that the light weighted in the blue region of the visible spectrum has the greatest influence on circadian rhythms (Brainard et al., 2008; Bullough, Bierman, Figueiro, & Rea, 2008; Cajochen et al., 2004; Lockley, Brainard, & Czeisler, 2003; Warman, Dijk, Warman, Arendt, & Skene, 2003). Studies examining light exposure in a natural setting have reported variability in illuminance level and blue spectral irradiance, particularly across seasons and age-groups (Thorne, Jones, Peters, Archer, & Dijk, 2009).

Research suggests that older people living in residential care homes may be exposed to very low levels of illuminance and spend short durations exposed to bright light. This has been attributed as a cause of circadian misalignment and irregularities in the sleep/wake profile (De Lepeleire, Bouwen, De Coninck, & Buntinx, 2007; Sinoo, van Hoof, & Kort, 2011). Shochat, Martin, Marler, and Ancoli-Israel (2000) reported the mean daytime light exposure in 66 institutionalized older adults was only 485 lux, determining this to be a very low illuminance level. Evidence suggests that the duration of exposure is also curtailed in those living in care homes. Ancoli-Israel, Clopton, Klauber, Fell, and Mason (1997) reported older people, aged 60–100 years, ranging in cognitive ability (i.e., mild, moderate, or no dementia) spent a median of only 9 min exposed to light above 1,000 lux. In the same study, the authors reported that people with more advanced cognitive decline (i.e., severe dementia) spent only 1 minute above 1,000 lux. Again, this is consistent with Shochat et al. (2000), reporting a median of 10.5 min in light of >1,000 lux. Although previous studies have documented both mean and median light levels and durations in particular light thresholds, the overall consensus remains that older people do not spend sufficient time in brightly lit environments (Ancoli-Israel, Clopton, Klauber, Fell, & Mason, 1997; Campbell, Kripke, Gillin, & Hrubovcak, 1988; Shochat, Martin, Marler, & Ancoli-Israel, 2000). A limitation of existing research is that light exposure was recorded during the spring or summer months when daylight is more plentiful. Insights into the differences in seasonal light exposure and specifically patterns of winter light exposure in older people are scant.

***… older people living in residential care homes may be exposed to very low levels of illuminance and spend short durations exposed to bright light***

Although insufficient illuminance levels have been attributed to poor sleep quality in older people, so too have other physiological functions (Van Someren, 2000). These included but are not limited to deterioration in the suprachiasmatic nucleus, the area of the brain where circadian rhythms are generated, cognitive ability, and the health of the visual system (Hughes & Neer, 1981; Swaab, Fliers, & Partiman, 1985; Van Someren, 2000). Typically, as cognitive ability declines, so too does sleep physiology, whereby people spend large periods of the day asleep and increased hours awake during the night (Amer, Harnza, El Akkad, & Abdel Galeel, 2013; Keage et al., 2012; Maggio et al., 2013; Wulff, Gatti, Wettstein, & Foster, 2010). In a cross-sectional study (*n* = 100), Amer, Harnza, El Akkad, and Abdel Galeel (2013) reported cognitive ability was negatively correlated with perceived poor sleep quality, although the association was small. Similarly, older adults may experience frequent nocturnal awakenings, difficulties falling asleep, and early awakenings during the morning hours (Cutler, Hughes, Singer, Sack, & Moffit, 1997; Espinosa-Fernandes, Cano-Lozano, & Miro-Morales, 1998; Maggi et al., 1998; Uchimura, Hirano, Mukai, Sakamoto, & Nakazawa, 1997).

It is now well established that the eye facilitates the “nonvisual” pathway to the circadian system in the brain and contains light-sensitive cells (intrinsically photosensitive retinal ganglion cells [ipRGC]; Hattar, Liao, Takao, Berson, & Yau, 2002). Research has also demonstrated that the eye changes with aging and is characterized by a narrowing pupil and yellowing lens (Hughes & Neer, 1981). These aging characteristics impede light transmission through the eye to vital nonimage-forming light-sensitive cells (Kessel, Lundeman, Herbst, Andersen, & Larsen, 2010). What is also understood is the ability for yellow/orange-tinted lenses to block blue light wavelengths, which happens naturally with increasing age (Sasseville, Paquet, Sevigny, & Hebert, 2006; Turner & Mainster, 2008). Blocking these vital blue light wavelengths may hinder circadian entrainment and contribute to a fragmented sleep pattern. Therefore, reduced light stimulus to the circadian system would indicate that lighting requirements are likely to differ across the life span (Turner & Mainster, 2008).

There is evidence to support a link between the level of cognitive ability and health of the visual system (Chang et al., 2014; Kondo, Niino, & Shido, 1994; Mandas et al., 2014; Nylén, Favero, Glimne, Teär Fahnehjelm, & Eklund, 2014). People living with cognitive decline (i.e., Alzheimer’s disease) are 3 times more likely to develop cataracts compared with healthy older adults and may be less likely to go outside (Van Someren, 2000). Furthermore, it has been reported in situations where cognitive decline has manifested incidences of cataract, macular degeneration, and deterioration in the light sensitive cells (ipRGC) are more frequent (Ancoli-Israel, Klauber, Gillin, Campbell, & Hoffstetter, 1994; Blanks, Torigoe, Blanks, & Hinton, 1996; Campbell et al., 1988; Paquet et al., 2007; Rastmanesh, 2011; Sanchez, Ge, & Zee, 1993).

This exploratory study is one of the first to present continuous objective measures of sleep/wake and light exposure patterns and measure visual function in older adults living in a residential care home setting. The aim of this research was to investigate, in two seasons, personal blue light exposure, illuminance levels, and health variables (i.e., sleep/wake patterns, daytime physical activity level, cognitive ability, and visual function). The following research questions were investigated:

**Research Question 1:** Do health and well-being outcomes (measured by sleep/wake, daytime physical activity, mental well-being, and cognitive ability) differ between seasons?
**Research Question 2:** Does light exposure (measured by blue light irradiance, illuminance level, and duration in thresholds) differ between seasons?

## Method

### Participants and Study Setting

Participants were recruited from six care homes across central Scotland. Potential participants were invited to attend coffee mornings to learn about the study. A visual aid was developed to communicate the study protocol and commitment required. Each participant gave written consent with additional written consent acquired from the next of kin. In total, 20 participants took part in the summer experiment (male = 3, female = 17) with 16 of these participants repeating the experiment in winter (male = 3, female = 13). The reduced participant numbers in winter were accounted for by one participant who passed away, two chose to withdraw from the process, and a lost data set for one further participant during download. For the purposes of the within-group analysis, the repeat 16 participants data sets were used. Study sites included (1) a refurbished Victorian period property (built between 1837 and 1901), with large glazed south facing communal areas (i.e., living and dining spaces) and bedrooms facing west; (2) a single story prefabricated home constructed during the 1970s, with communal spaces and bedrooms predominantly south facing; (3) three of the care homes were constructed in the early 1990s, communal rooms faced south and bedrooms west; and (4) a new build-assisted living facility completed in 2013. This was based on the model that residents had their own apartment (south or west facing) and the use of communal facilities, such as a function room, lounge, and dining area (south facing). The interior decor of all facilities was finished in a traditional pallet of colors and patterns, typically light colored walls and floor finishes if darker colored carpets.

### Apparatus

The sleep/wake cycle was monitored using the Philips Respironic Actiwatch. This is a wrist-mounted sensor (actiwatch), worn like a watch that records sleep/wake and activity movements. The actiwatch contains an accelerometer (or piezoelectric transducer) that produces a voltage in response to the changes in motion. Sleep is defined as the total activity count less than or equal to the wake threshold value, and wake and daytime physical activity is defined as total activity count greater than the wake threshold value. Mounted on the face of the actiwatch are light-sensitive photodiodes. These cells record illuminance levels in units of lux and blue spectral irradiances in units of (milliwatts per centimeter squared).

### Health and Well-Being Measures

The cohort was assessed using a range of health and well-being indicators. These were:

1. The Pittsburgh sleep quality index (PSQI) (Buysse, Reynolds, Monk, Berman, & Kupfer, 1989) assessed the sleep status over the last month. This is a self-rated measure consisting of 19 items generating a seven-component score (i.e., duration of sleep, sleep disturbance, sleep efficiency, daytime dysfunction due to lack of sleep, medication, etc.). The sum of these scores yields 1 global score, that is, total sleep quality. A score of <5 is classed as good sleep quality and a score of >5 is classed as poor sleep quality. The success of using this tool in an older demographic is supported by Amer et al. (2013), Valenza et al. (2013), and Nebes, Buysse, Halligan, Houck, and Monk (2009). These studies had sufficiently large samples sizes (minimum *n* = 100) reporting no limitations in use of this scale.
2. The mini mental state exam (MMSE) (Folstein, Folstein, & McHugh, 1975) was used as an initial screening for potential cognitive impairment. This is an 11-question measure comprising a number of different items, for example, participants were asked to repeat words and recall them at a later point in the assessment, counting down in increments of 7 from 100, spelling backward, and redrawing an image presented to them. The maximum possible score is 30. The cutoff points are defined as score 23–30 = normal, 19–22 = borderline, and <19 = potentially impaired. A score of <23 is considered as indicative of potential cognitive impairment. In this cohort, the mean MMSE score was 22 (*SD* = 6, in both seasons).
3. Deary–Liewald cognitive reaction time test (Deary, Liewald, & Nissan, 2011): This is a computer-based assessment conducted on a laptop computer. Participants were instructed to press any key to start the test. The computer screen presents a white box in the center, within which a black X appears. The aim was to press any key again when they saw the X appear to make it disappear. This was repeated for 20 counts and recorded in milliseconds.
4. Warwick Edinburgh Mental Well-Being Scale (Tennant et al., 2007): This is a 14-item scale that covers both hedonic (pleasure) and eudaimonic (meaning and self-realization) aspects of mental health. Typical questions include (a) *I’ve been feeling optimistic about the future*, (b) *I’ve been feeling cheerful*, (c) *I’ve been feeling loved,* (d) *I’ve had energy to spare,* or (5) *I’ve been interested in new things.* Participants rate these statements on a 5-point Likert-type scale with 1 = *none of the time* up to 5 = *all of the time* to create a single global score (maximum of 70). This scale has been successfully administered and is a reliable measure of well-being in older adults (Neale et al., 2016; Stewart-Brown et al., 2009).
5. *Visual function*—participants completed a set of visual function tests selected by ophthalmologists. The first assessment was the Bailey and Lovie (1976) LogMAR visual acuity test. This is a test of the sharpness of central vision and the ability to see fine details. The second measure was the Pelli–Robson test to measure contrast sensitivity (Pelli, Robson, & Wilkins, 1988). Contrast sensitivity is the visual ability to see objects that may not be outlined clearly or that do not stand out from their background. The visual assessments were conducted in situ. The test boards were placed at a distance of 3 m from the participant and in the same room at each measure. Participants read aloud the letters across and down the board until they could no longer decipher the letter. During summer, the mean room illuminance level (as measured by a spectrophotometer) was 146 lux and in winter 128 lux. The artificial light source was switched on, and luminaires were either compact fluorescent or halogen luminaires.

### Protocol and Study Design

A repeated measures design was used to explore sleep parameters and health and well-being outcomes in the sample between two seasons (summer and winter), in relation to environmental blue light exposure and illuminance levels. During the summer period (June–August 2014), weather conditions were favorable (daytime outdoor temperature range: 18°C–25°C, sunny with scattered cloud cover) and the winter period was typical for the study location (range: 5°C–10°C, heavier cloud cover and periods of rain). Sleep/wake and personal light exposure patterns were monitored for 4 days. Participants wore two body-mounted sensors: (1) wrist capturing sleep/wake and activity levels and (2) lapel capturing light exposure on the vertical plane more representative of light incident at the eye. The actiwatches sampled data at 15-s epochs. Each participant completed the cognitive assessments, mental well-being scale, and visual tests at the start of each wave of data collection.

## Data Analysis

Preset time intervals, as well as natural daytime light exposure, were based on previous literature (Shochat et al., 2000) and by a visual inspection of data sets. These include (1) morning 08:00–12:00, (2) natural waking day (set by the individual), and (3) evening 18:00–22:00. There was also the assumption made that most participants would typically be out of or in bed during these set intervals. The duration of time spent in illuminance threshold of >1,000 lux was investigated. These thresholds were derived from works by Bellia, Pedace, and Barbato (2013), Hubalek, Brink, and Schierz (2010), and Rea, Figueiro, Bierman, and Bullough (2010). Rea, Figueiro, Bierman, and Bullough (2010), Rea, Figueiro, Bullough, and Bierman (2005), and Rea, Figueiro, Bierman, and Hamner (2011) proposed a mathematical equation to derive a possible circadian stimulus (CS). Using the Rea model, Bellia et al. (2013) reported that the duration of time spent in a light threshold between 50 and 200 lux (at increasing increments of 50 lux) saw a significant increase in CS. However, it was stated that the exposure of duration in illuminance levels of 600 lux or above leads to a smaller increase in CS and at illuminance of greater than 1,000 lux saw a saturation in CS. Hubalek et al. (2010) similarly examined lux and spectral irradiance thresholds. They found that the exposure above 1,000 lux to be a significant predictor of sleep quality. The authors also stated that there was little information available to explore thresholds of spectral irradiance; therefore, the blue spectral irradiance threshold was arranged by a factor of 100, as carried out by Hubalek et al. (2010), thus a threshold of >100 μW/cm^2^ was investigated. In summary, there are three key light variables under exploration: (1) mean illuminance levels and blue spectral irradiances over a time period (morning, whole day, and evening), (2) durations spent in illuminance thresholds, and (3) durations spent in blue spectral irradiance thresholds. Data analysis was carried out using SPSS 21. Mean and median scores were computed for the suite of sleep/wake, light, and health and well-being assessments. Spearman’s ρ bivariate correlations were used to investigate associations between light and health variables by season. A within-group Wilcoxon signed-rank test was used to detect the changes over time.

## Results

Tables 1 and 2 summarize the associations between the health measures used in the study in each season. Relationships of interest in summer include MMSE positively associated with sleep onset latency (*r*s *=* .46, *p* = .039), that is, better cognitive performance was associated with a longer time to fall asleep. MMSE was negatively associated with visual acuity (right eye: *r*s = −.63, *p* = .003; left eye: *r*s = −.55, *p* = .011) and positively associated with contrasts sensitivity (*r*s = .58, *p* = .007), that is, poorer cognitive performance was associated with poorer visual acuity and poorer contrast sensitivity (Table 1). In winter, daytime physical activity was positively correlated with sleep efficiency (*r*s = .61, *p* = .011), that is, higher daytime activity levels were associated with higher sleep efficiency (Table 2). In summary, associations were found to be working as expected based on existing literature, that is, poorer cognitive performance was associated with poorer visual function and higher daytime physical activity was associated with better sleep efficiency. There were no significant associations between health and light exposures.

**Table 1.** Health Measures, Spearman’s ρ Bivariate Correlations (Summer).

Health and Well-being measures	WEMWBS	MMSE	SRT	PSQI	SOL	SE	WASO	TST	Wake Time	Bedtime	Physical Activity	CS Right	CS Left	VA Right	VA Left
WEMWBS	—														
MMSE	.09	—													
SRT	.11	−.46*	—												
PSQI	.18	.37	0.9	—											
SOL	.44	.46*	−.02	.46*	—										
SE	−.35	−.18	−.24	−.15	−.33	—									
WASO	.59**	−.05	.26	.14	.44*	−.43	—								
TST	−.01	.24	−.31	−.03	.11	.39	.11	—							
Wake time	.04	.44*	−.30	−.11	.10	.31	−.03	.37	—						
Bedtime	−.23	−.04	−.31	.18	−.20	.30	.15	.40	−.20	—					
Physical activity	.24	−.13	.18	.25	.03	.31	.11	−.16	−.73**	.05	—				
CS right	.21	.29	−.03	.28	.14	−.03	−.17	.14	.24	−.28	.11	—			
CS left	.50*	.58**	−.16	.33	.24	−.43	.23	.05	.45*	−.07	−.21	.49*	—		
VA right	−.08	−.63**	.16	−.23	−.11	.19	.15	−.13	−.49*	.21	.15	−.72**	−.68**	—	
VA left	−.44*	−.55*	−.16	−.31	−.37	.34	−.26	−.06	−.22	.12	.04	−.40	−.83**	.68**	—

*Note.* WEMWBS = mental well-being; MMSE = mini mental state examination; SRT = simple reaction time; PSQI = Pittsburgh sleep quality index; SOL = sleep onset latency; SE = sleep efficiency; WASO = wake after sleep onset; TST = total sleep time; CS = contrast sensitivity; VA = visual acuity.

*Correlation is significant at .05 (two-tailed).

**Correlation is significant at .01 (two-tailed).

**Table 2.** Health Measures, Spearman’s ρ Bivariate Correlations (Winter).

Health and Well-being measures	WEMWBS	MMSE	SRT	PSQI	SOL	SE	WASO	TST	Wake Time	Bedtime	Physical Activity	CS Right	CS Left	VA Right	VA Left
WEMWBS	—														
MMSE	−.01	—													
SRT	−.11	−.84**	—												
PSQI	.10	.19	−.11	—											
SOL	.19	.24	−.31	−.00	—										
SE	−.10	.13	−.10	.30	−.42	—									
WASO	.46	−.05	.15	−.15	.11	−.14	—								
TST	.42	.35	−.51*	−.14	.17	.05	.50*	—							
Wake time	−.20	.16	−.01	−.08	.02	.11	−.06	−.41							
Bedtime	.44	−.26	−.00	.08	.15	.06	.15	.45	−.57*	—					
Physical activity	.04	.19	−.32	.30	.02	.61*	−.24	.34	−.46	.31	—				
CS right	.10	.20	−.09	.12	−.20	−.22	−.17	−.25	−.14	−.10	−.23	—			
CS left	.29	.17	.00	.25	−.07	.99	.01	−.21	−.32	−.07	.00	.74**	—		
VA right	−.28	−.41	.32	−.32	−.13	.24	−.01	.03	.23	.07	−.01	−.73**	−.77**	—	
VA left	−.07	−.60*	.29	−.29	−.17	−.06	−.09	.07	−.02	.41	−.14	−.42	−.65**	.76**	—

*Note.* WEMWBS = mental well-being; MMSE = mini mental state examination; SRT = simple reaction time; PSQI = Pittsburgh sleep quality index; SOL = sleep onset latency; SE = sleep efficiency; WASO = wake after sleep onset; TST = total sleep time; CS = contrast sensitivity; VA = visual acuity.

*Correlation is significant at .05 (two tailed).

**Correlation is significant at .01 (two tailed).

### Seasonal Differences in Sleep/Wake, Activity, Mental Well-Being, and Cognitive Ability

**Figure 1.**
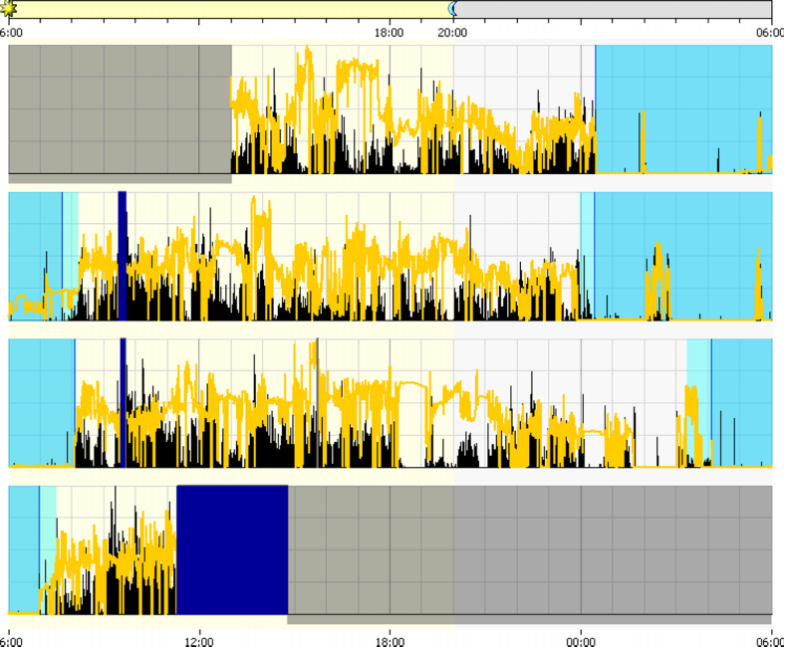
Actiwatch extract from summer. Black line denotes activity and yellow line denotes illuminance level.

**Figure 2.**
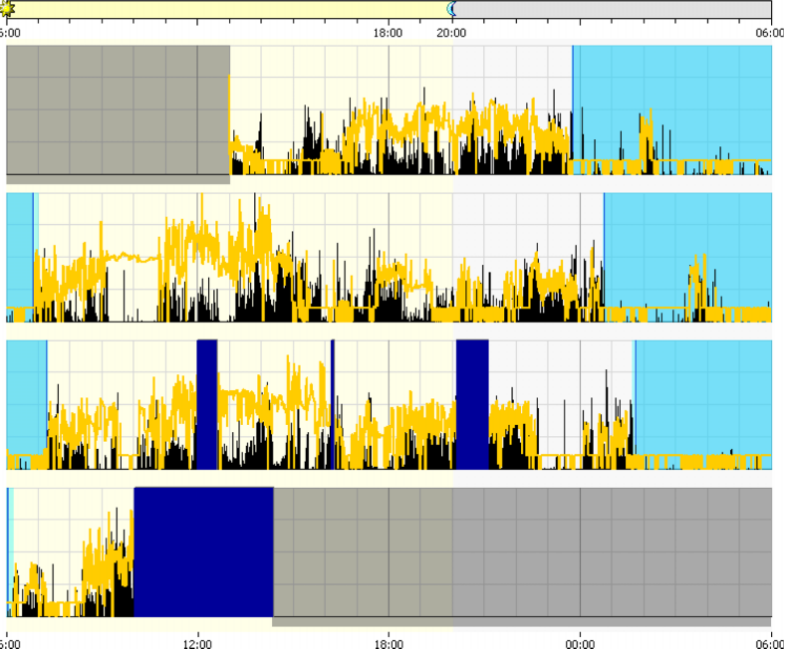
Actiwatch extract from winter. Black line denotes activity and yellow line denotes illuminance level.

Table 3 summarizes the within-group differences across sleep/wake, health, and well-being measures between seasons. Figures 1 and 2 illustrate differences in sleep/wake and activity patterns for summer (1) and winter (2). Daytime physical activity was significantly higher in summer (mean = 454 average active count) than in winter (mean = 174 average active count), indicating participants were more active during the summer season (*Z* = −3.51, *p* < .00). Results suggested a significant difference between visual acuity measured in the right eye between summer (mean = .39 logMAR) and winter (mean = .54 logMAR), indicating visual acuity decreased from the first measurement (*Z* = −2.44, *p* = .05).

**Table 3.** Differences in Health Measures, Mean (*SD*), and Wilcoxon Signed-Rank Test Across Seasons.

Health and Well-being measures	Summer	Winter	Wilcoxon Signed-Rank Test *(n = 16)*
	*M* (*SD*)	*M* (*SD*)	
Well-being			
WEMWBS	52 (9)	50 (11)	*z* = −.45, *p* = .64
Cognition			
MMSE	22 (6)	22 (6)	*z* = −.17, *p* = .86
SRT	811 (309)	740 (309)	*z* = −1.08, *p* = .27
Sleep/wake			
PSQI	6 (3)	6 (3)	*z* = −.53, *p* = .62
Onset latency	38 (43)	46 (34)	*z* = −.62, *p* = .53
Sleep efficiency	76 (10)	72 (14)	*z* = −.59, *p*= .55
WASO	53 (35)	68 (38)	*z* = −1.29, *p* = .19
Total sleep time	388 (119)	385 (139)	*z* = −.10, *p* = .98
Bed time	23:34 (2)	23:13 (2)	z=−.62, *p*= .53
Wake time	07:26 (1)	07:50 (2)	*z* = −.72, *p* = .46
Physical activity			
Active count/min	454 (143)	174 (43)	***z* =** −**3.51, *p* = .00, *r* =** −**.87**
Visual function			
CS right	1.07 (.39)	1.03 (.21)	*z* = −.81, *p* = .41
CS left	.93 (.47)	.90 (.47)	*z* = −.66, *p* = .50
VA right	.39 (.25)	.51 (.20)	***z* =** −**2.44, *p* = .01, *r* =** −**.61**
VA left	.54 (.49)	.52 (.49)	*z* = −.56, *p*= .57

*Note.* Statistical significance shown in boldface. WEMWBS = mental well-being; MMSE = mini mental state examination; SRT = simple reaction time; PSQI = Pittsburgh sleep quality index; WASO = wake after sleep onset; CS = contrast sensitivity; VA = visual acuity.

### Seasonal Differences in Blue Light Irradiance, Illuminance Level, and Duration in Thresholds

Table 4 summarizes the within-group differences across light exposures and durations spent in bright in light >1,000 lux and >100 μW/cm^2^ between seasons. Figures 1 and 2 illustrate differences in illuminance patterns for summer (1) and winter (2). Morning blue light exposure (*Z* = −3.00, *p* = .03) and morning illuminance level (*Z* = −2.98, *p* = .04) were both significantly higher in summer. Similarly, participants spent longer durations in bright light thresholds in summer compared to winter (*Z* = −2.91, *p* = .04). The corresponding blue light threshold of 100 μW/cm^2^ was also significantly different between seasons (*Z* = −2.66, *p* < .00). In summary, there was a significant difference in all light and threshold duration measures—including blue light—between seasons.

**Table 4.** Differences in Light Measures, Mean (*SD*), and Wilcoxon Signed-Rank Test Across Seasons.

Light exposures	Summer	Winter	Wilcoxon Signed-Rank Test *(n = 16)*
	*M* (*SD*)	*M* (*SD*)	
Morning illuminance (lux)	466 (894)	65 (59)	***z* =** −**2.89, *p* = .04, *r* =** −**.72**
Morning blue light (μW/cm^2^)	24 (57)	3 (4)	***z* =** −**3.00, *p* = .03, *r* =** −**.75**
Daytime illuminance (lux)	18 (41)	38 (42)	***z* =** −**2.99, *p* = .03, *r* =** −**.74**
Daytime blue light (μW/cm^2^)	360 (634)	51 (44)	***z* =** −**3.25, *p* = .04, *r* =** −**.81**
Evening illuminance (lux)	151 (2)	20 (15)	***z* =** −**3.15, *p* = .02, *r* =** −**.55**
Evening blue light (μW/cm^2^)	5 (8)	.5 (.5)	***z* =** −**3.36, *p* = .01, *r* =** −**.54**
Morn duration >1,000 lux	46 (56)	3 (8)	***z* =** −**2.91, *p* = .04, *r* =** −**.72**
Morn duration >100 μW/cm^2^	24 (33)	2 (0)	***z* =** −**2.66, *p* = .00, *r* =** −**.66**

*Note.* Statistical significance shown in boldface.

## Discussion

This study found statistical associations between cognitive ability (MMSE), visual function, sleep parameters, and daytime physical activity levels. Cognitive ability (measured by MMSE) was positively correlated with sleep onset latency and contrast sensitivity as well as negatively correlated with visual acuity. These relationships showed that better cognitive performance was associated with taking longer to fall asleep and better visual function. Daytime physical activity level was positively correlated with sleep efficiency. This showed that a higher daytime activity level was associated with better sleep efficiency. Results are consistent with existing evidence, reporting a decline in cognitive ability in older adults may be associated with a decline in the health of the visual system (Clemons, Rankin, & McBee, 2006; Dhillon & Lascaratos, 2009; Nylén et al., 2014; Salthouse, Hancock, Meinz, & Hambrick, 1996; Schmoll, Tendo, Aspinall, & Dhillon, 2011) and published findings reporting increased levels of physical activity in older adults may improve sleep quality (Alessi et al., 1995).

Objective measures of sleep patterns (e.g., total sleep time, sleep onset latency, wake after sleep onset, sleep efficiency, wake up time, and bedtime) or subjective sleep quality (PSQI) were not significantly different between seasons. Based upon the works of Kohsaka, Fukuda, Honma, Honma, and Morita (1992), Hubert, Dumont, and Paquet (1998), and Figueiro, Hamner, Higgins, Hornick, and Rea (2012), seasonal differences in sleep patterns (as measured by actiwatches) were expected to be more pronounced, for example, a phase shift in bedtime and wake time. Although a later bedtime and earlier wake time were present in this study, these were not significantly different between seasons. Reasons why there were no observed statistical differences in sleep outcomes could be due to the small sample size in this study or the mild nature of potential cognitive impairment (mean MMSE = 22) as more pronounced levels of sleep disruption may be more evident in older people with sever cognitive decline (MMSE score 0–9; Andersen, Wittrup-Jensen, Lolk, Andersen, & Kragh-Sørensen, 2004).

Between seasons, results suggested a significant difference in daytime physical activity level, that is, participants had higher daytime physical activity levels in summer compared to winter. This follows published work reporting humans are naturally more active in the summer months. Low levels of physical activity (or sedentary behavior) have previously been characterized as <100 active counts/min and suggest these are particularly low in older people living in residential care compared to those still residing in their own homes (van Alphen et al., 2016). In the current cohort, daytime physical activity levels were very low in winter and there was an absence of a physical activity program. To promote healthier sleep quality, action should be taken to reduce sedentary behavior, particularly during the winter period when older adults become less active.

***Between seasons, results suggested a significant difference in daytime physical activity level, that is, participants had higher daytime physical activity levels in summer compared to winter***

This exploratory study developed a useful protocol for measuring visual function and demonstrated it was possible to administer this in situ. For visual function outcomes, visual acuity (right eye) was significantly different between seasons, but contrast sensitivity was not, that is, visual acuity was poorer in winter. The need to determine the health of the visual system in future research is vital in understanding age-related changes in the transmission of light through the eye, the associated impacts on sleep, and the ability to design lighting schemes that respond effectively to nonvisual needs.

***The need to determine the health of the visual system in future research is vital in understanding age-related changes in the transmission of light through the eye, the associated impacts on sleep, and the ability to design lighting schemes that respond effectively to nonvisual needs.***

Light exposure was significantly different between seasons across all light measures, including morning and whole day for blue light and illuminance levels. This is consistent with Thorne, Jones, Peters, Archer, and Dijk (2009), reporting a significant difference in seasonal light across the daytime and blue spectral contribution of light. Overall, the natural illumination and blue light exposure patterns of this group were low (i.e., summer morning illuminance 466 lux and winter morning illuminance 65 lux). The mean duration of time above 1,000 lux for this study was 46 min during the summer and only 3 min in winter (a significant difference), that is, participants spent very few minutes in bright light conditions in both seasons. The duration of time spent in 1,000 lux or greater has been defined in previous studies as an indicator of time outdoors (Scheuermaier, Laffan, & Duffy, 2010). Illuminance readings and duration in light above 1,000 lux follow Shochat et al. (2000), who reported similarly low and short durations (mean daytime illuminance 485 lux and mean duration in light >1,000 lux to be 35 min). There remains ambiguity as to an ideal duration of exposure to bright light. Results here contribute to existing evidence by providing an important insight into the very short durations of time older people spent outdoors, particularly in the winter months. It is not uncommon for people (independent of age) to spend fewer minutes during the daytime in light >1,000 lux, particularly among those living at higher latitudes (Cole et al., 1995; Hubert, Dumont, & Paquet, 1998). Although the duration of exposure during the summer period is longer than previous reports, it is still a relatively limited amount of time and less than the duration healthy older adults may spend in bright light (Espiritu et al., 1994). Given the exploratory nature of this study, it is important to highlight specific limitations. Information in relation to other sleep confounders, such as medication use, nursing routine, diet choices, and/or life events (e.g., passing of other residents), was not documented. In follow-on studies, with larger numbers, collecting this information would help better understand sleep. Change in measure of visual acuity may be a genuine decline in visual functioning but could have been due to personal factors such as differing mood or alertness and concentration on the day of assessment, thus resulting in the participant reading the chart differently. Alternatively, unavoidable inconsistences such as sitting further away from the test chart, the angle of the test board, or differences in lighting quality may have contributed to change, although testing followed a standardized protocol. The following adaptation could help to improve consistency: an internally illuminated box chart would give a more consistent brightness for reading, limiting glare from the glossy surface of the chart, and maintaining a fixed angle at which to view the chart.

In conclusion, exposure to daylight (as measured by illuminance levels and blue spectral irradiance) was low as was time spent outdoors and daytime physical activity levels. This has important implications for the health and well-being of older care home residents, including sleep quality, visual function, and cognitive performance. The key findings of the study are the associations found between cognitive ability, sleep parameters, and visual function, and the low levels of light exposure independent of season. Designers of care home facilities should consider an architectural form and aperture design that encourages good daylight, offer seating and activity opportunities by windows, and facilitate easy transitioning to and walkability within outside spaces. Collectively, this research indicates the need to better understand the impact of the aging eye within the context of residential care, in order to generate lighting design guidance that is age appropriate going forward.

***Collectively, this research indicates the need to better understand the impact of the aging eye within the context of residential care, in order to generate lighting design guidance that is age appropriate going forward.***

## Implications in Practice

It emerged from this study that:

1. Older adults did not receive adequate light exposure in either summer or winter and actions should be taken to improve lighting provisions and facilitate time outdoors.
2. Participants were predominantly sedentary in winter, suggesting a need to increase activity levels in older adults living in a care home setting.
3. Lighting design studies should incorporate a measure of visual health to determine associations with circadian patterns and the efficacy of lighting provisions.
4. Architects and interior designers should consider how the design of the indoor environment could serve to promote physical activity.
5. Designers of care home facilities should consider an architectural form and aperture design that maximizes useful daylight illuminance, offers seating and activity by windows, and facilitates transitioning to outside spaces.

## Supplemental Material

Supplemental Material, Seasonal_Differences_in_Light_Exposure_and_the_Assocaitoins_with_Health - Seasonal Differences in Light Exposure and the Associations With Health and Well-Being in Older Adults: An Exploratory Study Click here for additional data file. Supplemental Material, Seasonal_Differences_in_Light_Exposure_and_the_Assocaitoins_with_Health for Seasonal Differences in Light Exposure and the Associations With Health and Well-Being in Older Adults: An Exploratory Study by Amanda Nioi, Jenny Roe, Alan Gow, David McNair, Peter Aspinall in HERD: Health Environments Research & Design Journal
