# Supplementary material for: Seasonal Differences in Light Exposure and the Associations With Health and Well-Being in Older Adults: An Exploratory Study
Source: HERD. 2017 Mar 15;10(5):64–79. doi: 10.1177/1937586717697650 (PMC5656103; doi:10.1177/1937586717697650)
Supplement: Supplemental Material, Seasonal_Differences_in_Light_Exposure_and_the_Assocaitoins_with_Health - Seasonal Differences in Light Exposure and the Associations With Health and Well-Being in Older Adults: An Exploratory Study [file Seasonal_Differences_in_Light_Exposure_and_the_Assocaitoins_with_Health.pdf]

HERD
HEALTH ENVIRONMENTS RESEARCH & DESIGN JOURNAL

THE CENTER FOR HEALTH DESIGN

"Seasonal Differences in Light Exposure and the Associations with Health and Well-Being in Older Adults: An Exploratory Study"

Authors: **Keys, Y., Silverman, E.**

Course ID # HERD58

Full Name	AIA # / EDAC #	Email	Phone #

Learning Objective 1: Explain the eyes 'nonvisual' role and how ageing determines the transmission of light through the eye to the circadian system.

Learning Objective 2: Explain the eyes 'nonvisual' role and how ageing determines the transmission of light through the eye to the circadian system.

Learning Objectives 3: Explain the key findings from the paper.

Learning Objective 4: Enumerate the implications for practice and suggest ways to translate this into design guidance.

Question 1: Describe the characteristics of the 'nonvisual' pathway, the ageing eye and how this affects the circadian system.

____

Question 2: Describe the characteristics of the 'nonvisual' pathway, the ageing eye and how this affects the circadian system.

____

Question 3: Give three examples of the implications for practice and suggest how you would apply one of these in practice.

a. ____

b. ____

c. ____

To earn AIA continuing education units please complete form, and email Jill Glaser at jglaser@healthdesign.org and your credits will be uploaded to your AIA account. To earn EDAC continuing education units please complete the form, retain the form for your records and self-submit the credits at the time of your EDAC renewal. The form is your "proof of attendance."
